# Knockout of the *HaREase* Gene Improves the Stability of dsRNA and Increases the Sensitivity of *Helicoverpa armigera* to *Bacillus thuringiensis* Toxin

**DOI:** 10.3389/fphys.2019.01368

**Published:** 2019-10-25

**Authors:** Ruobing Guan, Qiuyan Chen, Haichao Li, Shaoru Hu, Xuexia Miao, Guirong Wang, Bin Yang

**Affiliations:** ^1^State Key Laboratory of Wheat and Maize Crop Science, College of Plant Protection, Henan Agricultural University, Zhengzhou, China; ^2^Key Laboratory of Insect Developmental and Evolutionary Biology, Institute of Plant Physiology and Ecology, Shanghai Institutes for Biological Sciences, Chinese Academy of Sciences, Shanghai, China; ^3^State Key Laboratory for Biology of Plant Diseases and Insect Pests, Institute of Plant Protection, Chinese Academy of Agricultural Sciences, Beijing, China; ^4^Biobank of Ninth People’s Hospital, Shanghai Jiao Tong University School of Medicine, Shanghai, China

**Keywords:** *HaREase*, CRISPR/Cas9 system, RNAi efficiency, *Bt* resistance, dsRNA stability, insect immune

## Abstract

Double-stranded RNA (dsRNA)-induced genes are usually related to RNA interference (RNAi) mechanisms and are involved in immune-related pathways. In a previous study, we found a lepidopteran-specific nuclease gene *REase* that was up-regulated by dsRNA and that affected RNAi efficiency in Asian corn borer (*Ostrinia furnacalis*). In this study, to verify the function of *REase*, the homologous gene *HaREase* in cotton bollworm (*Helicoverpa armigera*) was knocked out using CRISPR/Cas9 system. We found that the midgut epithelium structure was apparently not affected in the Δ*HaREase* mutant [Knock out (KO)]. Transcript sequencing results showed that most of the known insect immune-related genes were up-regulated in KO. When second instar larvae were fed artificial diet with Cry1Ac, a protoxin from *Bacillus thuringiensis* (*Bt*), in sublethal doses (2.5 or 4 μg/g), the growth rate of KO was repressed significantly. The dsRNA stability was also enhanced in midgut extraction of KO; however, RNAi efficiency was not obviously improved compared with the wild type (WT). The KO and WT were injected with dsEGFP (Enhanced green fluorescent protein) and subjected to transcriptome sequencing. The results showed that the expression levels of 14 nuclease genes were enhanced in KO after the dsRNA treatment. These findings revealed that *HaREase* expression level was not only related with dsRNA stability, but also with *Bt* resistance in cotton bollworm. When *HaREase* was knocked out, other immune- or nuclease-related genes were enhanced significantly. These results remind us that insect immune system is complex and pest control for cotton bollworm is an arduous task.

## Introduction

RNA interference (RNAi) technology is a potential strategy for crop protection against insect pests by double-stranded RNA (dsRNA) spraying or feeding, or via transgenic plants (Nandety et al., 2015; Joga et al., 2016; Zhang J. et al., 2017; Whitten, 2019). However, RNAi efficiency is relatively low in lepidopteran insects and this is a core factor that frustrates the application of this technology (Terenius et al., 2011; Cooper et al., 2019). Many factors affect RNAi efficiency in insects, including the absence of RNA-dependent RNA polymerase (RdRP)-mediated synthesis of secondary small interfering RNAs (siRNAs) (Sijen et al., 2001), the rate of dsRNA processing into siRNAs (Guan et al., 2018a), the dsRNA uptake and transport mechanism (Luo et al., 2012), and the degradation rate of dsRNA (Christiaens et al., 2014; Spit et al., 2017; Song et al., 2019).

In a previous study, we identified an RNAi efficiency-related nuclease gene *REase* that was induced specifically by dsRNA from Asian corn borer (*Ostrinia furnacalis*). The REase protein had nuclease activity and could degrade various types of nucleic acids *in vivo* and *in vitro*. Overexpression of *REase* decreased the RNAi efficiency in *Drosophila*, and knocking down the expression level of *REase* improved RNAi efficiency in Asian corn borer (Guan et al., 2018b). Therefore, the expression levels of dsRNA-induced genes can influence RNAi efficiency. Further studies of dsRNA-induced genes will help to better understand the RNAi and immune-related pathways, and guide the theory and practice of using RNAi in pest control.

In this study, to explore the function of *REase* in other insects, we cloned the homologous gene of *HaREase* in cotton bollworm by PCR. Then, we used the CRISPR/Cas9 system to knockout the *HaREase* in cotton bollworm. We performed transcriptome sequencing, dsRNA degradation tests, and *Bacillus thuringiensis* (*Bt*) sensitivity bioassays, to determine the effect of *HaREase* on RNAi- and immune-related pathways. The results revealed that *HaREase* was a lepidopteran-specific gene that was expressed mainly in the midgut and was induced by dsRNA. The *HaREase* expression levels affected dsRNA degradation through changed other nucleases. We also discovered that *HaREase* was involved in the lepidopteran immune stress processes and affected the resistance of cotton bollworm to *Bt* toxicity. These results will provide a novel strategy to enhance the sensitivity of insects to *Bt* toxin by inhibiting immune-related genes. These results will provide a novel strategy to enhance the stability of insects to *Bt* toxin by inhibiting immune-related genes.

## Materials and Methods

### Insect Culture

Cotton bollworms were reared in the laboratory at 25°C and 75% relative humidity on a 14-h/10-h light/dark cycle. The larvae were fed a modified artificial diet (120 g of maize granules, 32 g of maize flour, 120 g of soybean flour, 4 g of vitamin C, 12 g of agar, 72 g of yeast powder, 4 g of sorbic acid, 60 g of glucose, 1.6 ml of formaldehyde, and 1000 ml of water). Moths were fed a 10% (v/v) honey solution.

### Single Guide RNA (sgRNA) Design and Synthesis

Ten individual cotton bollworms were used to identify of the conservative DNA fragment of the target *HaREase* gene by PCR with primers pairs (5′-GCCAAAGAAGAATCAAAGACTTATC AT-3′ and 5′-CCTCCTTGGCTTCAGTGACGCAATACC-3′). One small conservative sequence 5′-GCTGATGAAAGAT AGTCTCGCGG-3′ was selected as the target site and the corresponding sgRNA was synthesized using GeneArt^TM^ Precision gRNA Synthesis Kit (Thermo Fisher Scientific, Waltham, MA, United States). Basically, the target sequence of 5′-N20-3′ before PAM sequence “CGG” was used to design the sgRNA. A pair of long primers (5′-TAATACGACTCACTATAGCTGATGAAAGATAGT-3′ and 5′-TTCTAGCTCTAAAACCGAGACTATCTTTCATCA-3′) was designed and the sgRNA was synthesized following the manufacturer’s instructions. The Cas9 protein (GeneArt^TM^ Platinum TM Cas9 Nuclease) was obtained from Thermo Fisher Scientific.

### Microinjection and Screening of Mutants

Within half an hour of oviposition, the freshly laid eggs were collected and placed on a microscope slide and fixed with double-sided adhesive tape. Cas9 protein and sgRNA with final concentrations of 200 and 500 ng/μL, respectively, were microinjection into these pretreated eggs. Approximately 600 eggs were injected and incubated at 25°C and 75% relative humidity for 3–4 days until hatching. When these injected F0 individuals grew into adults, males and females were single paired with wildtype individuals in plastic cups for mating and laying eggs. The newly hatched F1 larvae were maintained into adults and single paired by inbreeding within same family. The laid-egg-adults of F1 were collected and their whole bodies were used for mutation screening by sequencing the genome DNA of each candidate individuals. If both of the F1 adults contain same mutant genotype, homozygous of mutants will be obtained in the F2 generation. The *HaREase* KO lines for functional analysis were obtained by inbreeding using F2 homozygous mutants. The genomic DNA was isolated using a TIANamp Genomic DNA Kit (Tiangen, Beijing, China). PCR products were purified using an EasyPure PCR Purification Kit (TransGen Biotech, Beijing, China), cloned into a pEASY-Blunt cloning vector (TransGen Biotech, Beijing, China), and sequenced for mutation screening.

### dsRNA Preparation

dsRNAs were synthesized using a MEGAscript RNAi kit (Ambion, Huntingdon, United Kingdom) in accordance with the manufacturer’s instructions. T7 promoter sequences were tailed to the 5′-ends of the DNA templates by PCR amplifications. All the primer sequences are listed in Supplementary Table S1. Template DNA and single-stranded RNA was removed from the transcription reaction by DNase and RNase treatments, respectively.

### *In vitro* Degradation of *HaREase* on dsRNA

Midgut extraction samples (Guan et al., 2018b) were collected from the fourth instar wild type (WT) and Δ*HaREase* mutant (KO), put them into 100 μl PBS, puncture and release the midgut fluid, take the supernatant after centrifugation. Dilute the midgut fluid with a final protein concentration of 3 ng/μl. After that, dsRNA was incubated with the diluted midgut fluid samples with a final concentration of 50 ng/μl at 37°C. The dsRNA samples were collected at different time points and the dsRNA integrity was analyzed by 1% agarose gel electrophoresis. The experiment was repeated four times.

### Sample Collection and RNA Isolation

Cotton bollworm samples were collected, immediately frozen in liquid nitrogen, and stored at −80°C until RNA extraction. Total RNA was isolated using TRIzol reagent (Invitrogen, Carlsbad, CA, United States) in accordance with the manufacturer’s instructions. Samples were treated with RNase-free DNaseI (New England Biolabs, Ipswich, MA, United States) for 30 min at 37°C to remove residual DNA.

### Real-Time Quantitative PCR (RT-qPCR)

Total RNA was extracted using TRIzol^®^ reagent (Invitrogen, Carlsbad, CA, United States) in accordance with the manufacturer’s instructions. First-strand cDNA was synthesized from 1 μg of RNA primed by oligo(dT)_18_ using M-MLV reverse transcriptase (Takara, Kyoto, Japan). The RT-qPCR assay for multiple genes was performed using SYBR^®^ Premix Ex TaqTM II (Takara, Kyoto, Japan). The primer sequences are listed in Supplementary Table S1. Melting curve analyses were performed for all of the primers. 18S rRNA expression levels were used to normalize the cycle threshold (Ct) (Chandra et al., 2014). The RT-qPCR was carried out using a Mastercycler ep realplex (Eppendorf, Hamburg, Germany). All the RT-qPCR assays were repeated three times. To assess the extent of RNAi, RNA was extracted from pools of three dsRNA-treated surviving larvae using TRIzol reagent (Invitrogen, Carlsbad, CA, United States), and each treatment was repeated three times. RT-qPCR reactions and data were analyzed in accordance with the methods of Livak and Schmittgen (2001). The data were analyzed using one-way analysis of variance to detect treatment effects compared with the untreated control.

### Transcriptome Sequencing and Analysis

The third instar larvae of WT and KO cotton bollworm were injected with dsEGFP (10 μg/larvae). Six hours later, samples were collected and frozen in liquid nitrogen for RNA sequencing (RNA-seq). Each sample contained 10 larvae. The non-treated samples were used as the control (WT). All the samples were sequenced using an Illumina HiSeq 2000 analyzer at BGI, Shenzhen, China. The datasets generated during the current study have been deposited in the NCBI Sequence Read Archive (SRA) under accession number PRJNA548442.

Sequences were searched against the GenBank non-redundant database (Nr) using the BLASTx algorithm. Gene ontology (GO) annotation was assigned using default annotation parameters in Blast2go^1^. The Cluster of Orthologous Groups (COG) and Kyoto encyclopedia of Genes and Genomes (KEGG) pathway annotations were assigned using the BLASTall online program against the respective databases^2, 3^. The RNA-seq expression levels were calculated using the reads per kilobase per million mapped read (RPKM) method.

### Light Microscopy

Tissue sectioning was performed as described by Fischer et al. (2008). Slides (5 μm) were stained using hematoxylin and eosin solution to show morphological changes. All images were taken using a fluorescent microscope (BX51, Olympus, Tokoyo, Japan) with differential interference contrast and the appropriate filter, unless otherwise stated.

### DNA Extraction and Microbial 16S rDNA Quantification

The midguts of third instar larvae were prepared and a DNeasy Blood and Tissue Kit (Qiagen) was used for DNA extraction. The ribosomal protein gene *RP*S3 (5′-GCCGCAGGATCCGTGAGCTG-3′ and 5′-ACCACGGGCA CCAGACTCCA-3′) was used as the internal reference, and the 16s rDNA (5′-TCCTACGGGAGGCAGCAGT-3′ and 5′-GGAC TACCAGGGTATCTAATCCTGTT-3′) was quantified by RT-qPCR.

### *Bt* Toxin and Bioassays

The Cry1AC protoxin was obtained from JZ Wei at Henan Agricultural University. Diet overlay bioassays (Hernandez-Rodriguez et al., 2008) were used to evaluate larval response to the *Bt* toxins. Cry1AC was diluted in 50 mM Na_2_CO_3_ (pH 10.0) and then added to the diet to give final concentrations of 2.5 μg or 4 μg Cry1AC/g diet. We used 12-well plates for the bioassay. Second instar larvae that hatched within 6 h were transferred onto the diet surface in each well. Each treatment was performed in triplicate (12 larvae in each plate was regarded as one repeat). After 3 days, the larvae were weighed and the statistical analysis was performed.

## Results

### Spatiotemporal Expression Patterns of *HaREase* in Cotton Bollworm

**FIGURE 1:**
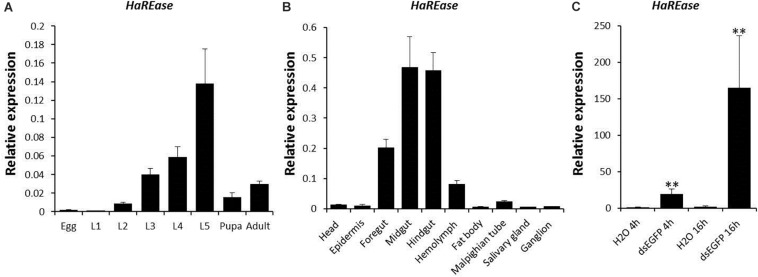
Expression patterns of *HaREase* in cotton bollworm by RT-qPCR. **(A,B)** Expression patterns of *HaREase* in different development stages **(A)** and different tissues **(B)**. L1–L5, First to fifth instar larvae. **(C)** Expression of *HaREase* after dsEGFP treatment for 4 and 16 h. Data are mean SD, *n* = 3. ^∗∗^*P* < 0.01.

The full-length 1737-nucleotide cDNA sequence of *HaREase* was cloned by PCR (XM_021337058.1). Then, the spatiotemporal expression levels of *HaREase* at eight development stages from egg to adult stages, and in 10 different tissues (head, epidermis, foregut, midgut, hindgut, hemolymph, fat body, malpighian tube, salivary gland, ganglion) were investigated by RT-qPCR (Figures 1A,B). The results indicated that *HaREase* was expressed mainly in the guts of larvae from the third to fifth instar larval stages. The expression pattern of *HaREase* in cotton bollworm was similar to that of *REase* in Asian corn borer. *HaREase* also was up-regulated by dsEGFP just as *REase* was in Asian corn borer (Figure 1C; Guan et al., 2018b). These results imply that *REase* genes may have similar functions in different insect species.

### Generation of *HaREase* Knockout Mutants of Cotton Bollworm

**FIGURE 2:**
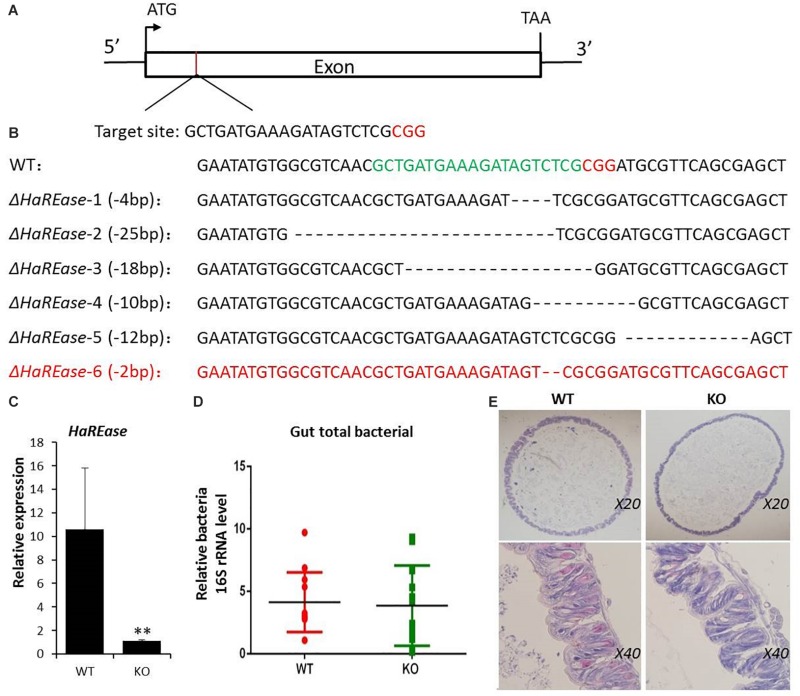
Targeted mutation of *HaREase* using the CRISPR/Cas9 system. **(A)** Schematic diagram of the sgRNA targeting site. The box represents the *HaREase* exon and the black line indicates the gene locus. The sgRNA target site is located on the sense strand of the exon. The protospacer adjacent motif (PAM) sequence is in red. **(B)** Sequences of the sgRNA target site in WT and six Δ*HaREase* mutants. The sgRNA target site sequence is in green and the PAM sequence is in red. **(C)** Confirmation of *HaREase* gene silencing in KO compared with WT. **(D)** Total numbers of bacteria in the midgut third instar larvae of WT and KO. **(E)** Hematoxylin and eosin stained images of the midgut of fifth instar larvae of WT and KO. Data are mean SD, *n* = 3. ^∗∗^*P* < 0.01.

The CRISPR/Cas9 system was used to knock out *HaREase* for further function analysis. sgRNA target site that covered the *HaREase* coding region was selected following the 5′-N20-NGG-3′ rule (Figure 2A). For the screening of mutants, six mutant lines with different genotypes were obtained in which Δ*HaREase*-1, Δ*HaREase*-2, Δ*HaREase*-4, and Δ*HaREase*-6 were effective mutants, but Δ*HaREase*-3 and Δ*HaREase*-5 were not because they did not break the ORF coding for the proteins. At last, the Δ*HaREase*-6 mutant line which lack two bases of nucleic acid sequences was selected for all the subsequent experiments (Figure 2B). Gene silencing of Δ*HaREase*-6 KO mutants was confirmed by RT-qPCR, which showed that the mRNA level of *HaREase* was significantly lower than in WT (Figure 2C). These results indicated that *HaREase* was effectively knocked out in this strian.

### Transcriptome and Intestinal Microorganism Analysis Between KO and WT Cotton Bollworm

**FIGURE 3:**
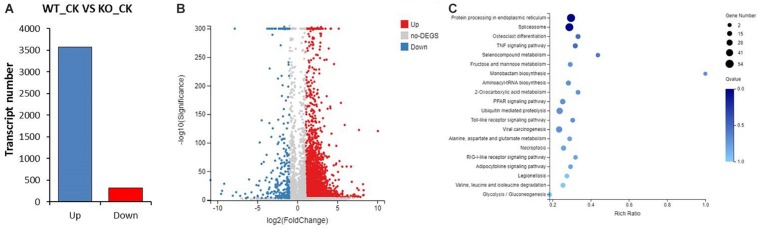
Analysis of the transcriptomes of KO and WT cotton bollworm. **(A)** Number of differentially expressed genes in KO compared with WT. **(B)** Volcano plot of the differentially expressed genes. **(C)** Top 20 KEGG pathways assigned to the differential genes (|LogFC| ≥ 1, FDR < 0.005, FPKM(WT_CK + KO_CK) ≥ 5).

We noticed that KO had no obvious visible differences in insect growth and development compared with WT. Because *HaREase* was expressed mainly in the gut of larval stage cotton bollworm (Figures 1A,B), we dissected out the midgut of fifth instar larvae of KO and WT individuals to look for anatomical differences. No obvious differences were observed (Figure 2E). Furthermore, analysis of the 16s rDNA from the intestinal microorganism of third instar larvae by RT-qPCR found no significant effect on intestinal flora of KO compared with WT (Figure 2D). To investigate the effect of *HaREase* knock out on other genes, we performed transcriptome sequencing analysis of the third instar larvae midguts of WT and KO. Compared with WT, 3574 transcripts were up-regulated in KO (LogFC > 1, FDR < 0.005) and 325 transcripts were down-regulated (LogFC < −1, FDR < 0.005) (Figures 3A,B). The KEGG pathway analysis of these differentially expressed genes identified spliceosome, protein processing in endoplasmic reticulum, ubiquitin mediated proteolysis, and mRNA surveillance as the top metabolic pathways associated with the knockout of *HaREase* (Figure 3C). Four immune-related pathways were further calculated, unexpectedly, most of the immune-related genes were up-regulated in KO strain (Table 1).

**TABLE 1:** Expression patterns of immune-related genes in WT and KO cotton bollworm.

**Gene ID**	**WT_CK FPKM**	**KO_CK FPKM**	**Nr**
110370534	1.04	7.3	XP_021182050.1|0.0e+00| CWF19-like protein 1
110373764	2.2	15.2	XP_021186788.1|0.0e+00| nuclear factor NF-kappa-B p110 subunit
110378270	15.66	43.63	XP_021193249.1|0.0e+00| putative ATP-dependent RNA helicase Pl10 isoform X2
110380251	10.05	22.14	XP_021195851.1|3.6e-192| stimulator of interferon genes protein-like isoform X3
110370227	306.59	938.38	XP_021181649.1|1.8e-194| activating transcription factor of chaperone isoform X2
110371527	4.65	10.16	XP_021183524.1|1.2e-171| caspase-1
110377308	2.14	9.12	XP_021191820.1|0.0e+00dynamin-1-like protein isoform X3
110377385	1.19	4.11	XP_021191948.1|2.3e-207| cyclic AMP response element-binding protein A
110380242	0.73	5.3	KPJ02490.1|5.0e-240| Stress-activated protein kinase JNK
110380325	3.07	11.06	XP_021195949.1|8.3e-302| TNF receptor-associated factor 1
110382611	1.41	4.09	XP_021198936.1|4.3e-262| TGF-beta-activated kinase 1 and MAP3K7-binding protein 1-like
110372021	13.1	37.2	XP_021184199.1|1.3e-106| ras-related protein Rac1
110374863	1.29	6.12	XP_021188437.1|3.2e-229| myeloid differentiation primary response protein MyD88-like
110379776	0.85	6.6	XP_021195254.1|4.1e-156| toll-interacting protein B-like
110371163	1.87	7.52	XP_021182954.1|2.4e-119| charged multivesicular body protein 4b
110371187	7.74	25.49	XP_021182997.1|0.0e + 00| FAS-associated factor 1 isoform X2
110371859	0.82	5.15	XP_021183962.1|3.9e-302| high mobility group protein DSP1-like
110374555	5.23	11.48	XP_021187983.1|4.3e-104| charged multivesicular body protein 1b
110375638	4.88	14.54	XP_021189504.1|7.9e-252| vacuolar protein sorting-associated protein 4A
110380382	11.21	23.76	XP_021196024.1|1.1e-132| apoptosis-inducing factor 1
110383764	13.64	155.03	XP_021200313.1|0.0e+00| protein ref(2)P-like isoform X1

### *HaREase* Knockout Enhanced the Sensitivity of Cotton Bollworm to *Bt* Toxin

**FIGURE 4:**
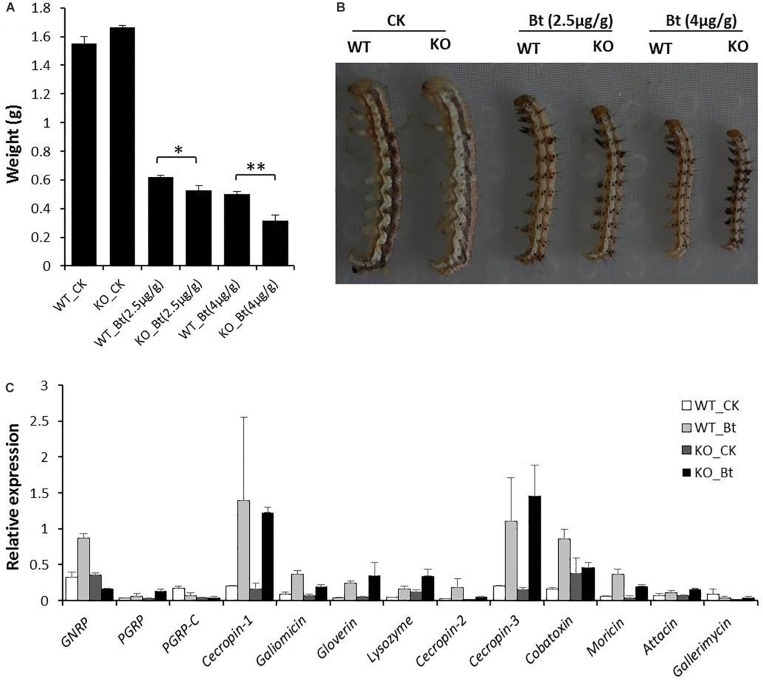
Sensitivity of KO and WT cotton bollworm to *Bt* toxins. **(A,B)** Growth and development of KO and WT cotton bollworm after feeding with an artificial diet containing Cry1Ac. **(C)** Expression levels of immune-related genes in KO and WT after feeding with Cry1Ac. Data are mean SD, *n* = 3. ^∗^*P* < 0.05; ^∗∗^*P* < 0.01.

To understand the effects of the up-regulated immune-related genes on cotton bollworm, the larvae were treated with sublethal doses of *Bt* toxin. Second instar larvae were fed an artificial diet containing the Cry1Ac protoxin in sublethal doses of 2.5 or 4 μg/g. Three days later, the larvae were weighed, and the weight gain of the KO larvae was significantly lower than that of the WT larvae (Figures 4A,B). This indicated that *HaREase* may be involved in insect immunity or detoxification of Cry1Ac.

After feeding with Cry1Ac, the expression levels of several immune related genes were detected, c*ecropin-1*, *galiomicin*, *gloverin*, *lysozyme*, *ceropin-3*, *cobatoxin*, and *moricin* were up-regulated significantly in both WT and KO (Figure 4C), whereas GNRP and *cecropin-2* were up-regulated only in WT. This may be related to difference in Cry1Ac resistance between KO and WT strain (Figure 4C).

By comparing the expression levels of *Bt* receptor and resistance-related genes between WT and KO, we detected one *Bt* receptor gene and three genes encoding resistance-related enzymes that were down-regulated significantly in KO (Table 2). Three other *Bt* receptor genes were also up-regulated significantly in KO, especially the major *Bt* receptor gene that encodes aminopeptidase N (Table 2). These results may explain why KO was more sensitive to *Bt* toxin than WT, and indicate *HaREase* as a new candidate gene for tackling the *Bt* resistance in cotton bollworm.

**TABLE 2:** Expression levels of *Bt* receptor genes and *Bt* resistance-related genes in WT and KO cotton bollworm.

**Gene ID**	**WT_CK FPKM**	**KO_CK FPKM**	**Nr**	**Annotation**
110379591	147.93	62.48	AKH49599.1|0.0e+00| alkaline phosphatase 2	Bt receptor
110378012	282.02	1245.85	AAK85539.1|0.0e+00| aminopeptidase N	Bt receptor
110372809	9.24	32.72	XP_013145187.1|0.0e+00| ATP-binding cassette sub-family B member 6	Bt receptor
110380022	14.13	60.2	XP_021195552.1|0.0e + 00| protocadherin Fat 1-like	Bt receptor
110379025	136.29	19.64	XP_021194194.1|8.5e-138| trypsin CFT-1-like	Bt resistance-related gene
110383548	154.1	74.82	XP_021200017.1|1.1e-148| trypsin CFT-1-like isoform X3	Bt resistance-related gene
110379026	1858.48	582.65	XP_021194195.1|0.0e+00| transmembrane protease serine 9-like	Bt resistance-related gene
110380580	111.6	242.22	XP_021196268.1|4.6e-149| trypsin, alkaline C-like	Bt resistance-related gene
110379569	217.63	939.5	XP_021194961.1|8.6e-158| chymotrypsin-1-like	Bt resistance-related gene
110380581	9.8	40.53	XP_021196270.1|6.0e-149| trypsin CFT-1-like	Bt resistance-related gene

### Effect of *HaREase* on dsRNA Stability and the RNAi Pathway

**FIGURE 5:**
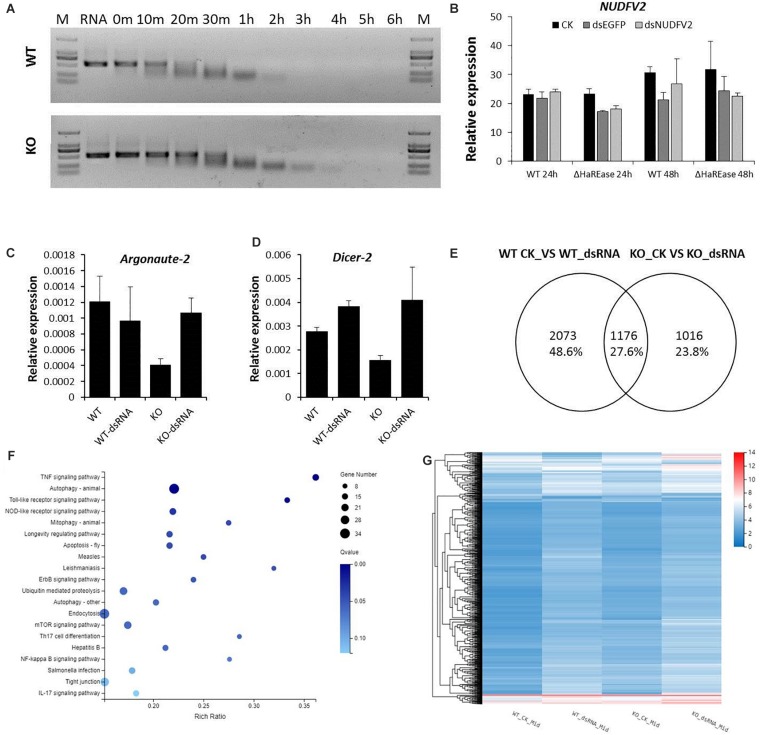
Effects of *HaREase* on the dsRNA degradation rate and RNAi pathway. **(A)** Degradation of dsRNA in midgut extractions form KO and WT. Diluted KO and WT midgut extraction solutions were incubated with 50 ng/μl dsEGFP. The dsRNA degradation rate was detection by agarose gel electrophoresis. **(B)** RNAi efficiency of 10 μg dsNUDFV2 injected into the third instar of WT and KO larvae. The expression levels of *NUDFV2* were detected by RT-qPCR 6 h after treatment. **(C,D)** Expression levels of *argonaute-2* and *dicer-2* after dsEGFP treatment in the third instar of WT and KO larvae. **(E)** Veen diagram of dsEGFP-induced differentially expressed genes in WT and KO. **(F)** KEGG pathway analysis of the differentially expressed genes. **(G)** Heat map showing differences in gene expression between WT and KO. The color scale indicates the different expression levels.

To further confirm the effect of *HaREase* on dsRNA stability, the midgut fluid was extracted from the fouth instar larvae of WT and KO. dsRNA was incubated in the midgut extract and agarose gel electrophoresis was used to detect the degradation rate of the dsRNA. The result showed that dsRNA degraded more quickly in the midgut extract from WT compared with the midgut extract from KO (Figure 5A). *NUDFV2* was selected to test RNAi efficiency between WT and KO. The same amount of ds*NUDFV2* was injected into third instar larvae of WT and KO. After 6 h, samples were collected and the expression level of *NUDFV2* was checked. However, the RNAi efficiency of dsNUDFV2 was not enhanced obviously in KO (Figure 5B). Next, we checked the expression levels of the core RNAi pathway genes *argonaute-2* and *dicer-2* by RT-qPCR and found that both these genes were up-regulated in KO after dsRNA treatment, but no significant differences in their expression levels were detected between WT and KO after the dsRNA treatments (Figures 5C,D). These results indicated that *HaREase* knockout had no obvious effect on RNAi efficiency in cotton bollworm, and are completely different from what was found in Asian corn borer (Guan et al., 2018b). This suggests that the RNAi mechanism in cotton bollworm as a polyphagous insect is more complicated and variable than in Asian corn borer.

To further clarify the relationship between *HaREase* and RNAi efficiency, the third instar cotton bollworm larvae of WT and KO were injected with dsEGFP (10 μg/larvae). After 6 h, the samples were collected for transcriptome sequencing. The results indicated that many genes had changed after dsEGFP treatment, among them, 1176 transcripts were co-changed genes (Figure 5E). The KEGG annotations indicated that these genes were involved mainly in spliceosome, protein processing in endoplasmic reticulum, and ubiquitin mediated proteolysis pathways (Figure 5F). The heat map showed that the expression levels of some of the transcripts were significantly different between the WT and KO samples (Figure 5G).

We thoroughly analyzed the nuclease genes in WT and KO and found that 12 of 15 nuclease genes were up-regulated in KO before and after dsRNA treatment compared with their expression in WT (Table 3). The increased expression of these nucleases in KO may explain why the RNAi efficiency of cotton bollworm was not enhanced by knocking out *HaREase*.

**TABLE 3:** Expression levels of nuclease genes in WT and KO cotton bollworm before and after dsRNA treatment.

**Gene ID**	**WT_CK**	**WT_dsRNA**	**KO_CK**	**KO_dsRNA**	**log2(KO_CK/WT_CK)**	**log2(KO_dsRNA/WT_dsRNA)**	**Nr**
110370696	8.51	2.14	1.41	0.92	–2.593463969	–1.21790503	Three prime repair exonuclease 2-like
110381609	0.79	12.66	1.88	8.07	1.250808104	–0.649636826	Endoribonuclease Dicer isoform X2
110375907	34.41	74.01	94.44	91.11	1.456570149	0.299889193	Exonuclease GOR-like isoform X1
110370058	0.34	2.8	0.94	4.29	1.46712601	0.615550821	DIS3-like exonuclease 2
110383072	2.08	7.35	6.4	7.37	1.621488377	0.003920369	Flap endonuclease 1
110380131	0.43	2.71	1.34	3.05	1.639824436	0.170516391	RNA exonuclease 4
110376593	1.06	5.17	4.07	6.38	1.94096453	0.303392143	Exonuclease 3′-5′ domain-containing protein 2
110374975	0.27	4.21	1.14	3.8	2.078002512	–0.147820815	5′-3′ exoribonuclease 1 isoform X1
110383848	2.05	8.01	9.49	16.78	2.210784178	1.066868568	Double-stranded RNA-binding protein Staufen homolog 2 isoform X3
110378878	1.18	10.71	5.5	15.5	2.220644759	0.533309735	Exosome component 10
110371061	2.78	12.72	13.18	13.98	2.245193582	0.13626569	Endoribonuclease LACTB2
110383990	0.98	3.88	5.34	6.38	2.445986088	0.717499772	Exosome complex exonuclease RRP44
110370590	0.35	5.66	2.44	6.28	2.801454321	0.149962506	Fanconi-associated nuclease 1-like
110377349	0.52	6.09	4.32	12.85	3.054447784	1.077254226	5′-3′ exoribonuclease 2 homolog
110378912	0.27	1.88	2.53	8.36	3.228106073	2.15277028	Poly(U)-specific endoribonuclease homolog

## Discussion

The KO mutants that we obtained using CRISPR/Cas9 technology were used to study the function of *HaREase* in cotton bollworm. The results indicate that *HaREase* is a lepidopteran-specific gene that can be induced specifically by dsRNA and is expressed mainly in the gut of cotton bollworm at the larval stage. The *HaREase* mutation had no obvious effect on the growth and development of cotton bollworm, but KO was more sensitive to Cry1Ac than WT. The results suggest that *HaREase* may be associated with insect immunity and will be a promising new target for tackling the *Bt* resistance in cotton bollworm.

A primary concern is the impact of *HaREase* mutations on the RNAi efficiency of cotton bollworm. Many results have indicated that the nucleases used for dsRNA degradation can affect RNAi efficiency. Knock-down of *dsRNase* in the *Leptinotarsa decemlineata* midgut enhanced RNAi efficiency (Spit et al., 2017). Our previous research indicated that the expression level of *REase* was related to RNAi efficiency in Asian corn borer (Guan et al., 2018b). Therefore, we tested the dsRNA degradation rate in midgut extractions from WT and KO and found that although dsRNA stability was enhanced in KO, the RNAi efficiency was not improved obviously (Figure 5).

RNA interference efficiency depends on the dsRNA degradation rate by insect nucleases (Wang et al., 2016; Song et al., 2019). Nuclease activity has been detected in *Culex pipiens quinquefasciatus* (Calvo and Ribeiro, 2006), *Glossina morsitans* (Caljon et al., 2012), *Nezara viridula* (Lomate and Bonning, 2016), *Bombyx mori* (Arimatsu et al., 2007), and *Schistocerca gregaria* (Wynant et al., 2014). Many researches have revealed that RNAi efficiency in different insect orders species was highly correlated with dsRNA stability (Wang et al., 2016; Song et al., 2019). The dsRNA was more stable in coleopteran insects than in lepidopteran insects and RNAi efficiency was higher in coleopteran than in lepidopteran (Ivashuta et al., 2015). Therefore, knocking down nuclease activity using RNAi technology can enhance RNAi efficiency (Spit et al., 2017; Guan et al., 2018b; Song et al., 2019). For instance, *dsRNase* knockdown enhanced RNAi efficiency in *L. decemlineata* (Spit et al., 2017). However, this does not work in all insect species, and *dsRNase* knock down did not enhance RNAi efficiency in *S. gregaria* (Spit et al., 2017). The diversity of nucleases in different insects may be the main reason for the observed differences in RNAi efficiency. In this study, the transcriptome sequencing results confirmed that when one nuclease gene *HaREase* was knocked out, the expression levels of a large number of other nuclease genes were enhanced to compensate. Therefore, at least for cotton bollworm, it is difficult to increase RNAi efficiency by knocking out only one nuclease gene.

When exogenous dsRNA is introduced into the insect body, it can be recognized as a pathogen-associated molecular pattern (PAMP) by pattern-recognition receptors (PRRs) to induce an immune response (Sadler and Williams, 2008). Indeed, the RNAi pathway is a natural antiviral immunity system (Ding, 2010; Gammon and Mello, 2015; Meki et al., 2018). The RNAi pathway related genes *argonaute-2*, *dicer-2* and the immune-related genes *hemolin*, *attacin*, *gloverin*, *lysozyme*, and *NF-kappa B* are induced by dsRNA (Hirai et al., 2004; Guan et al., 2018c). Because *HaREase* is expressed mainly in insect gut and can be induced by dsRNA, we speculated that *HaREase* may be related to the insect immune response. Knockdown of an immune-related gene in *Spodoptora littoralis* increased its susceptibility to *Bt* toxin (Caccia et al., 2016). When we combined *Bt* toxin with dsRNA targeting chymotrypsin-like genes, larval mortality increased and inset death was accelerated (Guan et al., 2016). Thus, a new strategy for enhancing the impact of *Bt* toxins on insects may be the knocking down of immune-related genes. When sublethal doses of Cry1Ac were added into the artificial diet, the KO mutants were more sensitive to *Bt* and their weight gains decreased significantly (Figures 4A,B), indicating the expression level of *HaREase* was related to insect sensitivity to *Bt* toxin. Therefore, *HaREase* may be a new target for improving *Bt* sensitivity in cotton bollworm.

*Bt* toxins from *B. thuringiensis* have been used as an insecticide in cotton bollworm, which has mitigated the impact of the destructive cotton bollworm pest worldwide (Sanahuja et al., 2011; Romeis et al., 2019). When *Bt* protoxins are eaten by insects, they can be digested by midgut proteases into activated toxins that bind to receptors in the insect midgut, forming lytic pores in the membrane and leading to cell breakdown and insect death (Gill et al., 1992; Bravo et al., 2011). However, the evolution of insect resistance to *Bt* is gradually reducing the benefits of this approach (Tabashnik and Carriere, 2017). Many factors are related to *Bt* sensitivity, including the *Bt* receptors and the proteases involved in protoxin activation (Rajagopal et al., 2009; Cao et al., 2013; Liu et al., 2014; Jin et al., 2018). For example, the reduced binding capacity of *Bt* toxins to midgut receptors such as like ALP, APN, cadherin, and ABCC2 (Xu et al., 2005; Jurat-Fuentes and Adang, 2007; Gahan et al., 2010; Baxter et al., 2011; Atsumi et al., 2012; Xiao et al., 2014; Chen et al., 2015; Zhang T. et al., 2017), and the reduced expression levels of trypsin genes, and *ALP2* are all related to *Bt* resistance in cotton bollworm (Wei et al., 2018). In this study, we identified three *Bt* receptor genes that were enhanced significantly in KO (Table 2). Besides this, after feeding with a diet containing Cry1Ac, the expression levels of the immune-related genes *GNRP*, *PGRP-C*, *cecropin-1*, *galiomicin*, *cecropin-2*, *cobatoxin*, *moricin*, and *gallerimycin* were lower in KO than in WT (Figure 4C). Together, these results suggest that *HaREase* is involved in insect immune stress response against *Bt* toxicity.

The insect immune response against various external invasions is a complex process, and some immune genes are lepidopteran-specific. *Hemolin* is a lepidopteran-specific gene that encodes a pattern recognition protein that can be induced by bacteria and viruses (Aathmanathan et al., 2018). *HaREase* is also lepidopteran-specific gene (Guan et al., 2018b), study these lepidopteran specific genes will help in understanding the specific immune pathway of these insects and contribute to the management of cotton bollworm.

## Data Availability Statement

The datasets generated during the study have been deposited in the NCBI Sequence Read Archive (SRA) under accession number PRJNA548442.

## Author Contributions

RG and QC performed the experiments. RG and BY designed and wrote the manuscript. HL and SH analyzed the data. XM and GW revised the manuscript. All authors read and approved the manuscript.

## Conflict of Interest

The authors declare that the research was conducted in the absence of any commercial or financial relationships that could be construed as a potential conflict of interest.

## Footnotes

1 http://www.blast2go.com

2 http://www.ncbi.nlm.nih.gov/COG

3 www.genome.jp/kegg
